# Anticoagulant Treatment in Severe ARDS COVID-19 Patients

**DOI:** 10.3390/jcm11102695

**Published:** 2022-05-10

**Authors:** Adrian Ceccato, Marta Camprubí-Rimblas, Elena Campaña-Duel, Aina Areny-Balagueró, Luis Morales-Quinteros, Antonio Artigas

**Affiliations:** 1 Critical Care Research Center, Institut d’Investigació i Innovació Parc Taulí I3PT, ParcTaulí, 08208 Sabadell, Spain; mcamprubi@tauli.cat (M.C.-R.); ecampana@tauli.cat (E.C.-D.); aareny@tauli.cat (A.A.-B.); luchomq2077@gmail.com (L.M.-Q.); aartigas@tauli.cat (A.A.); 2 CIBER of Respiratory Diseases (CIBERES), Institute of Health Carlos III, 41092 Madrid, Spain; 3 Bioscience and Medicine Faculty, Universitat Autònoma de Barcelona, Bellaterra, 08193 Catalunya, Spain; 4 Department of Intensive Care Medicine, Hospital Universitari Sant Pau, 08025 Barcelona, Spain

**Keywords:** COVID-19, heparin, nebulization, inflammation, coagulation

## Abstract

Patients with COVID-19 may complicate their evolution with thromboembolic events. Incidence of thromboembolic complications are high and also, patients with the critically-ill disease showed evidence of microthrombi and microangiopathy in the lung probably due to endothelial damage by directly and indirectly injured endothelial and epithelial cells. Pulmonary embolism, deep venous thrombosis and arterial embolism were reported in patients with COVID-19, and several analytical abnormal coagulation parameters have been described as well. D-dimer, longer coagulation times and lower platelet counts have been associated with poor outcomes. The use of anticoagulation or high doses of prophylactic heparin is controversial. Despite the use of anticoagulation or high prophylactic dose of heparin have been associated with better outcomes in observational studies, only in patients with non-critically ill disease benefits for anticoagulation was observed. In critically-ill patient, anticoagulation was not associated with better outcomes. Other measures such as antiplatelet therapy, fibrinolytic therapy or nebulized anticoagulants are being studied in ongoing clinical trials.

## 1. Introduction

The infection by severe acute respiratory syndrome coronavirus 2 (SARS-CoV-2) may cause severe disease in some patients. In patients with the most critically-ill disease, severe pneumonia with acute respiratory distress syndrome (ARDS) is usually presented and thromboembolic complications are common. Severe extra-pulmonary complications have been described as well.

The incidence of venous thromboembolic events in patients with critically-ill disease by COVID19 could be higher than 22% [1], and controversies about prophylactic dose of heparin have raised. 

## 2. Pathophysiology

SARS-CoV-2 infects the host cells through binding the angiotensin-converting enzyme 2 (ACE2) receptor, present in alveolar type II epithelial cells, bronchial epithelial cells and endothelial cells [2]. 

It is well known that sepsis activate cytokine cascade and activate coagulation factors [3], but COVID-19 also has shown direct viral infection of the endothelial cell and diffuse endothelial inflammation [4]. Unlike influenza, COVID-19 causes 9 fold-times more alveolar capillary microthrombi, microangiopathy and more angiogenesis [5]. Diffuse alveolar damage was common in both diseases. In a study published by Lax and col [6], the authors found in autopsies of patients who died by COVID-19, the presence of obstruction of pulmonary arteries by thrombotic material present at both the macroscopic and microscopic level. These findings were associated with lung parenchymal bleeding and hemorrhagic infarction. Moreover, pulmonary infarction was complicated by bronchopneumonia in some cases.

Pulmonary increased proinflammatory cytokines are ascribed to damaged alveolar epithelium and endothelium and cellular apoptosis, activation of alveolar macrophages, recruitment of proinflammatory cells in the alveolar compartment and lymphocytes apoptosis. Extensive inflammation of the endothelium may cause multiple organ dysfunction [2,7,8]. 

**Figure 1:**
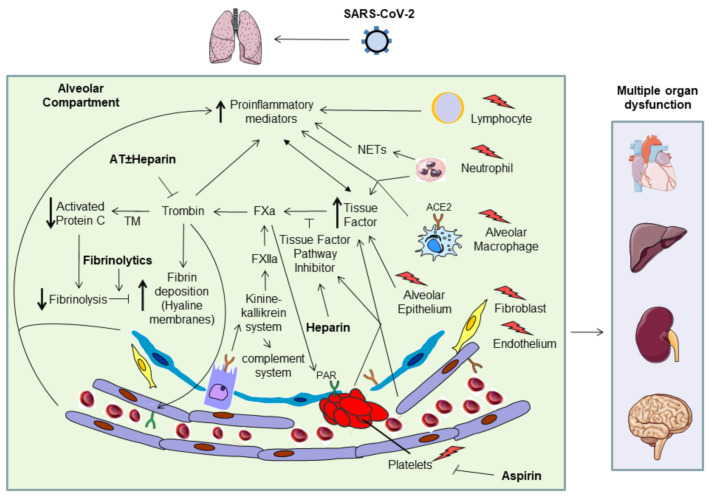
Coagulation and inflammatory pathway active by SARS CoV2.

Injured endothelial cells release von Willebrand factor (vWF), that promote platelet aggregation, and Angiopoietin-2, which in turn binds its receptor Tie2 and abrogates Angiopoietin-1 binding, leading to vasoconstriction and tissue factor (TF) expression [9]. TF is also expressed on alveolar epithelial cells, macrophages/monocytes, neutrophils and platelets, and its activation leads to extrinsic coagulation cascade activation and fibrin formation [9,10]. In addition, proinflammatory cytokines are potent activators of TF through the transcription of NF-kB, a protective mechanism that pretends to limit blood loss and avoid the spreading of foreign particles from the initial point of infection. Activated neutrophils further enhance coagulation through the release of damage-associated molecular patterns (DAMPs) and neutrophil extracellular traps (NETs), composed of DNA, cytotoxic histones and neutrophils elastase, which accumulate in the microvessels of patients with severe COVID-19 and induce microthrombosis [9] (Figure 1).

## 3. Thrombotic Events

Nopp and col [1] reported in a meta-analysis including 66 studies in COVID-19 patients a prevalence of venous thromboembolism (VTE) of 14%. This prevalence may change from 9 to 40% if ultrasound was used as diagnostic method. In the same study the prevalence of VTE for patients admitted to ICU was 22% for VTE, 13% for pulmonary emboli (PE) and 18% for deep venous thrombosis (DVT). Patients with VTE were predominantly male and had higher d-dimer measures at admission.

Chi and col [11] analyzed in other meta-analysis the incidence of VTE in patients who received thromboprophylaxis with heparin; Twenty-three percent of patients developed at least one episode of VTE. A higher incidence of VTE (30%) was found in patients admitted to ICU, compared with a 13% of incidence in general ward. Higher incidence of PE but not of DVT was found between patients admitted to ICU and those admitted to general ward. 

In another meta-analysis, Tufano and col [12] evaluate the risk difference between patients with or without COVID-19. Compared to patients without COVID-19, patients with COVID-19 have a 6% higher risk to develop a VTE event, in particular in patients admitted to ICU.

The incidence of arterial thrombotic events was 3.7% in patients admitted to ICU in 3 Dutch ICUs [13]. Stroke, lower limb ischemia, and myocardial injury have been described in patients with COVID-19. Ischemic stroke has been described in patients without risk factors and the cause of myocardial injury is unknown and seems related with direct viral injury. In a single center study from Japan, the authors found that 48% of thrombotic events were arterial [14]. In other study with active search for arterial thrombotic events, the odds ratio for arterial thrombosis in COVID-19 was 3.37 [15].

The risk for thrombotic events is increased after discharge as well, the incidence of VTE after discharge was 1.8 according to reported by Zuin and col. Age and male was associated with higher risk of VTE after discharge [16]. 

On the other side, hemorrhagic complications with mayor bleeding were observed in 5% of patients [17]. Patients with COVID-19 may present criteria for DIC with a higher mortality (88%) [18].

Abnormal coagulation parameters were associated with worst prognosis, higher D-dimer and fibrin degradation product levels, longer prothrombin time (PT) and activated partial thromboplastin time (aPTT) were found more commonly in patients who did not survive [19]. Coagulation factors, protein C and antithrombin were lower in patients who did not survive, even though levels keep within normal range. D-dimer was independently associated with higher risk of thrombotic events [20,21]. Reduced fibrinolytic activity was found in patients admitted to ICU compared with non ICU admitted patients [22]. A study evaluating thromboelastography showed a hypercoagulable profile characterized by mean Alpha angle, extremely low LY30 values and higher fibrin contribution toward clot formation relative to platelets [17]. Another study evaluating thromboelastography showed a state of hypercoagulability as shown by increased values of K angle and MA (which define the velocity of clot formation and the maximal amplitude of the clot, respectively) [23]. The presence of lupus anticoagulant were described as well mainly in patients with prolonged aPTT [24]. 

Lower platelet count was associated with more severe disease, and thrombocytopenia was associated with higher risk of death [25,26], also lower increase in the number of platelets from day 1 to day 3 was independently associated with death in a multicenter Spanish study [27].

## 4. Anticoagulant Treatment

The higher incidence of thrombotic events has brought controversies about the optimal dose of prophylactic heparin and measures to prevent these events. Anticoagulant or heparin (low molecular weight (LMWH) or unfractioned (UFH)) prophylactic-intensity, intermediate-intensity and therapeutic-intensity have been proposed for the management of patients without evidence of VTE [28]. 

UFH is naturally released by intestinal or lung mast cells, basophils in the blood and endothelial cells [29,30] and after chemical or enzymatic depolymerisation of UFH, LMWH is produced [31]. Both UFH and LMWH increase the inhibitory activity of various serine protease inhibitors, such as antithrombin (AT). Nevertheless, because of the depolymerization process, only UFH raises the inhibitory activity of AT for thrombin. 

Furthermore, anticoagulants have been proposed as treatment for severe critically-ill conditions that shared similar pathophysiology, with hypercoagulability and inflammatory disorders such as sepsis and ARDS [29,32].

Early initiation of prophylactic anticoagulation compared with no-anticoagulation showed a decreased risk of death of 27% (hazard ratio (HR) 0.73 95% confidence interval (95% CI) 0.66 to 0.81) in an observational study from USA with 4297 patients [33]. The benefit was stronger in patients not admitted to ICU and the risk of major bleeding was low. In another observational study only including patients admitted to ICU, early therapeutic anticoagulation was not associated with better outcomes, with a HR for mortality of 1.12 (95% CI, 0.92 to 1.35) [34].

Standard dose of LMWH could not achieve accepted prophylactic levels of antiXa. In an observational study levels of antiXa were lower with standard dose compared with intermediate dose [35]. Nevertheless, intermediate dose was evaluated in an open label randomized controlled trial compared with standard dose in patients admitted to ICU, showing no benefit for intermediate dose [36]. In this study, 600 patients were randomized, and 562 included in the pre specified analysis. The primary outcome was a composite outcome including: acute VTE, arterial thrombosis, treatment with extracorporeal membrane oxygenation (ECMO) or all-cause mortality within 30 days of enrollment. No differences between groups for the primary outcome were observed (45.7% vs. 44.1% OR 1.06 (95% CI 0.76 to 1.48)). No differences were neither observed in secondary outcomes (all-cause mortality, VTE, ventilator free days). Major bleeding complications were similar between groups and severe thrombocytopenia was most common in the intermediate dose group. 

Levels of d-dimer have been proposed for categorized patients who may benefit from higher doses of anticoagulants. In a randomized clinical trial of 253 adults with d-dimer levels 4-fold-times upper limits or sepsis-induced coagulopathy, standard, intermediate or therapeutic anticoagulant doses were evaluated. Patients who received therapeutic dose presented less incidence of the primary outcome (death, arterial or venous thromboembolic events). This reduction of incidence was driven by reduction in VTE and no difference in mortality was observed. No differences in the analysis for the primary outcome were found in patients admitted to ICU at randomization [37]. RAPID a randomized adaptive clinical trial evaluated the effectiveness of therapeutic doses of LMWH in moderately ill patients and increased d-dimer levels [38]. A significant reduction in mortality was observed in patients receiving therapeutic anticoagulation (1.8% vs. 7.6%, OR 0.22 95% CI 0.07 to 0.65).

In a multiplatform study integrated by Antithrombotic Therapy to Ameliorate Complications of COVID-19 (ATTACC), Accelerating COVID-19 Therapeutic Interventions and Vaccines-4 Antithrombotics Inpatient Platform Trial (ACTIV-4a) and the Randomized, Embedded, Multifactorial Adaptive Platform Trial for Community-Acquired Pneumonia (REMAP-CAP), two trials evaluated in critically ill and moderately ill patients with COVID-19, standard vs. therapeutic doses of LMWH [39,40]. One-thousands two hundred seven critically ill patients were randomized, and no differences in the organ-support free days were observed. The study was stopped by met futility criteria. Fifty-one percent of patients included in standard care received intermediate doses. The OR for in-hospital mortality was 0.84 (95% credible interval, 0.64 to 1.11; posterior probability of inferiority, 89.2%).

For non-critically ill patients results were opposed. The study was stopped early because criteria for the superiority of therapeutic-dose anticoagulation were met. In this study, the posterior probability that therapeutic-dose anticoagulation increased organ support–free days as compared with usual-care thromboprophylaxis was 98.6% (median adjusted OR, 1.27; 95% credible interval, 1.03 to 1.58). Moreover, the posterior probabilities that patients in the therapeutic-dose anticoagulation group were more likely to survive without organ support (99.1%). 

In a RCT evaluating standard prophylactic dose compared with therapeutic anticoagulation with rivaroxaban (or heparin in unstable patients), patients admitted to the hospital and high d-dimer levels showed similar results in terms of time to death, duration of hospitalization or duration of supplemental oxygen to day 30 [41]. 

Results of meta-analyses published showed controversial results, therapeutic anticoagulation doses may be better for non-critically ill patients, without benefits for critically ill patients [42,43,44]. 

Side events and bleeding were not high in the clinical trials performed. In the RAPID study [38], bleeding was lower in patients with therapeutic dose compared with prophylactic dose. In the multiplatform study [39], for critically ill patients major bleeding events occurred in 3.8% of patients assigned to therapeutic doses. In the HEP-COVID trial [37], the number needed to harm in the overall population was 33, while in the non-ICU stratum, the number needed to harm was approximately 2000. 

## 5. Other Antithrombotic Treatments

Several non-anticoagulants treatments have been tested in patients with COVID-19 (Table 1). 

Antiplatelet therapies have been tested in patients with COVID-19 as well. Aspirin is a non-selective inhibitor of the cyclooxygenase pathway, known to decrease platelet recruitment and inflammation. In hospitalized patients, aspirin did not reduce the 28-day mortality, however increase the chance to be discharged alive at 28 days [45]. In other study in non-critically ill patients hospitalized for COVID-19 the use of a P2Y12 inhibitor in addition to a therapeutic dose of heparin, did not result in an increased odds of improvement in organ support-free days within 21 days during hospitalization [46].

In patients with severe COVID-19, the use of fibrinolytic therapy with tissue plasminogen activator may improve pulmonary outcomes. In a retrospective study with 79 patients, the use of alteplase was associated with improvements in oxygenation parameters [47]. Alteplase showed to be safe in a phase 1–2 study with 50 randomized patients. None severe bleeding occurred and also improvement in oxygenation was observed [48].

## 6. Nebulized Anti-Coagulants

COVID-19 is a complex disease regarding its pathophysiology with a positive and uninterrupted feedback loop between inflammation and coagulation systems, with a special attention to the pulmonary compartment, since COVID-19 multiorgan dysfunction focused in the lung. Considering the importance of the pulmonary compartment with a proinflammatory and procoagulant state, nebulized anti-coagulants administration directly to the lung by nebulization might allow higher dosages, could increase their local efficacy and avoid systemic complications produced by intravenous coagulation administration.

The most severe cases of COVID-19 could be benefited of being treated with nebulized heparin thanks to its anticoagulant, anti-inflammatory, antimicrobial and antiviral actions.

In patients with ARDS, controversial results have been found while administering local heparin. Nebulized heparin (50,000 IU/day, 100,000 IU/day, 200,000 IU/day and 400,000 IU/day) did not produce adverse effects and reduced pulmonary coagulopathy in patients with ARDS [49,50]. In a randomized phase 2 study nebulized heparin (25,000 IU) decreased the days of mechanical ventilation in ARDS patients [51], and in a phase 3 clinical trial (CHARLI) nebulized heparin (250,000 IU) every 6 h to day 10 was well tolerated with decreased lung injury progression and earlier return at home in patients with invasive ventilation [52]. In contrast, in a multicenter randomized controlled trial with nebulized heparin focused on heparin safety and efficacy in burn patients with inhalation trauma (HEPBURN) increased systemic clotting times and adverse events were found and the trial was stopped for safety [53]. 

No convincing benefit of heparin nebulization was found under mechanical ventilation [54] nor for prophylaxis for pneumonia patients receiving mechanical ventilation [55,56]. In 16 patients with VILI, heparin was nebulized proving safety and increasing the number of ventilator-free days [57].

Nebulized antithrombin (AT) and/or heparin in in vivo models of ALI decreased pulmonary coagulation and inflammation, but did not alter systemic coagulation nor bleeding [58,59]. Although heparin competes with syndecan-4 for binding AT diminishing the coagulant-independent anti-inflammatory effect of AT, the anti-inflammatory effect of AT through coagulant mediators and the anti-inflammatory effects of heparin are maintained. The activated pathways by AT and heparin might be different according to the dose, time and route of administration, whose might have an effect on their interaction.

Nebulized anti-coagulants could be an alternative in patients with COVID-19 [7]. In an uncontrolled study, nebulized heparin was administered in 98 patients with COVID-19, the main outcomes were aPTT level at baseline and the peak after nebulization. Increase of aPTT was significantly higher after nebulization, however this difference seems clinically irrelevant (34 s to 38 s in patients receiving LMWH and 78 s to 84 s in patients receiving UFH). An exploratory analysis showed a significantly increases in oxygenation parameters after nebulization was started [60]. Several ongoing interventional studies are evaluating whether nebulized heparin may improve outcomes in patients with COVID-19 [61,62,63]. 

## 7. Recommended Management

**Figure 2:**
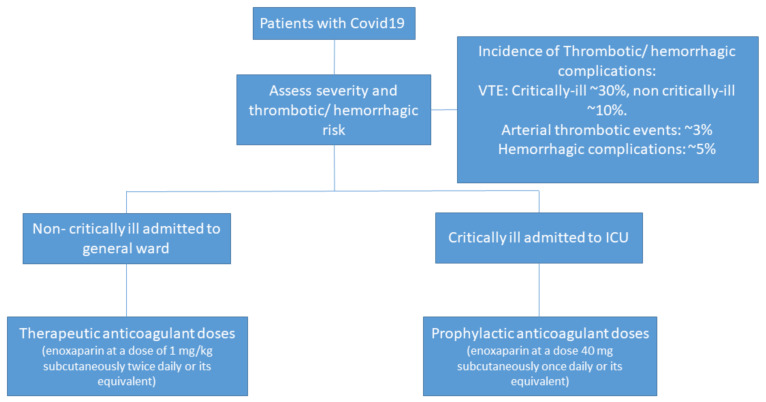
Initial clinical management. Abbreviations: ICU = Intensive care unit; VTE = venous thromboembolism.

Patients admitted by COVID-19 should be monitored for thromboembolic events. Those patients with abnormal coagulation parameters, lower platelets count or higher d-dimer have higher risk of thrombotic events and worst outcomes. Patients with non-critically-ill diseases should receive anticoagulation dose of heparin during acute phase or until discharge. Rivaroxaban 10 mg/daily could be administered after discharge in patients with increased risk for venous thromboembolism during 35 days after discharge according to MICHELLE trial [64]. Patients with critically-ill disease should be treated with prophylactic dose of heparin until discharge or during acute phase according to local standard of care. A summary of clinical management is presented in Figure 2.

It is unknown what should be done when patients have an impairment in their clinical condition and became critically-ill. Seems adequately change from full dose to prophylactic dose if thrombotic event is not suspected. 

In case of major bleeding or major adverse events, the risk and benefits of therapeutic/prophylactic doses of anticoagulants should be evaluated in each case.

## 8. Conclusions

Incidence of thrombotic events is increased in patients with COVID-19. Several direct and indirect mechanism are involved in the pathophysiology of thrombotic events. Prophylactic dose of heparin is recommended in patients with critically-ill disease and anticoagulation dose in patients with non-critically-ill disease. Several therapeutic agents are being evaluated in ongoing clinical trials.

Further studies are warranted to evaluate the impact of the use of immunomodulatory drugs, antivirals and antithrombotic drugs in clinical practice. Moreover, when similar pathophysiology patterns could be involved in ARDS or pneumonia caused by other pathogens different from coronavirus.

## Figures and Tables

**Table 1:** Other antithrombotic treatments.

Treatment	Safety and Benefit
Antiplatelet therapy	None benefit on mortality, Aspirin may improve probability of been discharged alive at 28-day.
Fibrinolytic	Safety proved in phase 1–2, benefits on oxygenation have been observed.
Nebulized anticoagulants	Nebulized heparin showed safety profile. Benefits on oxygenation were observed in patients with moderate COVID-19.

## Data Availability

Not applicable.
